# Measuring the impact of suppression on visual acuity in children with amblyopia using a dichoptic visual acuity chart

**DOI:** 10.3389/fnins.2022.860620

**Published:** 2022-07-15

**Authors:** Bixia Zhu, Meng Liao, Longqian Liu

**Affiliations:** ^1^Department of Optometry and Visual Science, West China Hospital, Sichuan University, Chengdu, China; ^2^Department of Ophthalmology, West China Hospital, Sichuan University, Chengdu, China

**Keywords:** amblyopia, suppression, visual acuity, dichoptic eye chart, mean luminance

## Abstract

**Purpose:**

To develop a novel dichoptic visual acuity chart that measures the impact of interocular suppression on the visual acuity of each eye when two eyes are open.

**Methods:**

Fifty-four subjects (19 anisometropic amblyopia, 20 treated amblyopia, and 15 normal children) participated in this study. The visual acuity that was tested under dichoptic-optotypes condition (i.e., presented optotypes to the untested eye) was compared with that under monocular condition (i.e., cover the untested eye with opaque patch). Visual acuity differences between these two conditions were compared among the three groups. The correlations between visual acuity differences and the depth of interocular suppression were then computed. Some participants performed the visual acuity test under dichoptic-luminance condition (i.e., presented mean luminance to the untested eye), and the test-retest reliability was established.

**Results:**

A reduced visual acuity of the non-dominant eye was found in the dichoptic-optotypes condition for the amblyopia group (*P* < 0.001) and the treated group (*P* = 0.001); the difference in the treated group was less than that in the amblyopia group (*P* < 0.001) but more than that in the normal group (*P* = 0.026). A significant correlation was found between the visual acuity differences and the depth of suppression, which was tested with a binocular phase combination task (*P* = 0.005). No change was found in the dichoptic-luminance condition.

**Conclusion:**

The amblyopic eye and the previous amblyopic eye seem to suffer from a reduced visual acuity when two eyes are open due to suppression. This was successfully captured by our novel and reliable dichoptic-optotypes visual acuity chart.

## Introduction

Amblyopia is a neurodevelopmental disorder that results from poor visual development during the critical period. Symptoms include poor monocular visual acuity and impaired binocular function. The rate of amblyopia in the general population is 1–4% (Multi-ethnic Pediatric Eye Disease Study, 2008; Williams et al., 2008; Multi-Ethnic Pediatric Eye Disease Study, 2009); the number of individuals with amblyopia might be 221.9 million by 2040 (Fu et al., 2019). In unilateral amblyopes, there is an imbalanced suppression of visual input between the eyes; for example, the suppression from the fellow eye to the amblyopic eye is stronger than the one originating from the amblyopic eye to the fellow eye, thereby creating an imbalance (Huang et al., 2011; Zhou et al., 2018). Studies indicate this form of imbalanced suppression between the eyes in amblyopia determines both monocular and binocular visual functions (Hess and Thompson, 2015). However, the standard means to diagnose amblyopia is measuring the lines of logMAR difference in visual acuity between the eyes (Wallace et al., 2018). Also, monocular visual acuity of the tested eye is usually tested while the untested eye is occluded. However, the monocular occlusion minimizes interocular interaction (Lai et al., 2012; Jia et al., 2015).

It is believed that imbalanced suppression between the eyes perturbs the visual acuity of the amblyopic eye. This has been shown in previous studies. For instance, Pugh (1954) used the orthoptoscope to present test dots (subtending different angles equivalent to 1/60–6/6 Snellen letter) to the amblyopic eye and fixation dot to the fellow eye. By changing the luminance of the fellow eye via neutral density filters, Pugh showed that the acuity of the amblyopic eye decreased as the light level of the fellow eye increased. Moreover, von Noorden and Leffler (1966) also found that the visual acuity of the strabismic amblyopic eye was worse when there was visual input in the fellow eye. von Noorden and Leffler (1966) used polaroid filters to present the visual acuity chart to the amblyopic eye, but a black chart surface to the fellow eye. Lai et al. (2011) and Lai et al. (2012) showed that the visual acuity of the amblyopic eye was reduced when it was partially patched (by a square patch that occluded the central visual field of the fellow eye) compared to when it was fully patched. Nevertheless, the relationship between the content of reduced visual acuity in the amblyopic eye under the dichoptic condition and the depth of suppression seems to remain opaque.

Recovery of amblyopia is often determined by tracking the difference in visual acuity between the eyes after a period of monocular treatment. However, studies show that individuals who have been supposedly treated with amblyopia as measured with their improved visual acuity of the amblyopic eye still exhibit binocular imbalance as a function of spatial frequency (Chen et al., 2017, 2021; Zhao et al., 2017). This finding indicates that the binocular imbalance, which could be due to imbalanced suppression, in amblyopia still remains even if visual acuity gets improved throughout standard treatment such as monocular occlusion of the fellow eye (Kehrein et al., 2016; Jia et al., 2018; Chen et al., 2019). If suppression plays a primary role and an impaired visual acuity is merely a subsequent event due to suppression (Li et al., 2011; Hess et al., 2014), residual binocular imbalance indicates that the current method of treatment for amblyopia is inadequate to ensure a full recovery of the visual function. Also, to what content the binocular imbalance perturbs the visual acuity of the previous amblyopic eye while the fellow eye receives visual input is still unclear.

To answer our question, we designed a dichoptic visual acuity chart. This chart has two new features. First, suppression has been found to exhibit dependence on spatial frequency (Kwon et al., 2015; Mao et al., 2020), and that it can be influenced by interocular contrast (Birch et al., 2019) or luminance (Zhou et al., 2013) difference. In other words, the presentation of stimuli at different spatial frequencies, contrast, or luminance between both eyes might introduce interocular imbalance that might otherwise be absent. However, in our study, we presented the untested eye at the same spatial frequencies (i.e., the same size of optotypes), contrast (i.e., 100% Weber Contrast) and luminance as the tested eye. Second, the optotypes shown to the two eyes were vertically arranged and were not perceived as being overlapped; this feature is in contrast as those used in previous studies where overlapping optotypes were used to test suppression at various interocular contrast ratios (Kwon et al., 2015; Birch et al., 2016). In these studies, optotypes presented to two eyes should be of low spatial frequencies so that subjects can see them clearly. When it comes to a visual acuity test (higher spatial frequency), overlapping arrangement could produce confusion to the observer.

We found that the amblyopic eye and the previous amblyopic eye had reduced visual acuity when the fellow eye was viewing optotypes rather than mean luminance. The magnitude of visual acuity change in dichoptic and monocular conditions was correlated with the depth of suppression. Our new dichoptic letter chart demonstrated a robust test-retest reliability. Therefore, we recommend that the dichoptic visual acuity chart be used to measure the visual acuity of amblyopes in the future.

## Materials and methods

### Participants

Fifty-four children were enrolled in the ophthalmology department of the Western China Hospital, Sichuan University: 15 normal individuals (9.27 ± 2.19 years old; mean ± SD), 19 anisometropic amblyopes (8.95 ± 2.97 years old), and 20 treated amblyopes (8.00 ± 2.73 years old). All participants underwent comprehensive clinical examinations, including previous treatment history, best-corrected visual acuity (BCVA), slit-lamp examination, ophthalmoscopic exam, stereoacuity, alignment exam, and extraocular muscle movements. BCVA was tested using a Tumbling E Logarithmic Visual Acuity Chart (xk100-06, China). Stereoacuity was tested with the TNO stereogram (TNO 18th, Lameris Ootech BV, Celsiusbaan 6B, 3439 NC, Nieuwegein, the Netherlands). This study adhered to the tenets of the Declaration of Helsinki and was approved by the ethics committee of the Western China Hospital. Written informed consent was obtained from the patients’ guardians or parents.

Amblyopia was diagnosed according to the Preferred Practice Pattern of The American Academy of Ophthalmology (Wallace et al., 2018). Individuals were classified as having anisometropic amblyopia if they had an interocular BCVA difference greater than 2 lines, or interocular BCVA difference less than 2 lines but the amblyopic eye’s visual acuity worse than 0.1 logMAR, with anisometropia greater than 1.50 D in spherical lens or 1.00 D in cylinder lens. Treated amblyopia was defined as a BCVA of the previous amblyopic eye achieving 0.1 logMAR and an interocular acuity difference of less than 2 lines. The normal controls had a normal BCVA (≤ 0.1 logMAR), no risk factors (i.e., strabismus, uncorrected anisometropia), and no history of amblyopia. Patients were excluded from this study if they had a history of organic eye disease and had undergone patching or cycloplegia within 4 h just before the measurement of our experiments. The clinical details of the participants are provided in Supplementary Table 1.

In this report, we refer to the amblyopic eye of the amblyopia group, the previous amblyopic eye of the treated group, and the non-acuity dominant eye (i.e., the eye with worse BCVA) (Coren and Kaplan, 1973; Vedamurthy et al., 2007) of the normal group as the non-dominant eye (NDE) and the other eye as the dominant eye (DE).

### Apparatus

The dichoptic visual acuity test and the binocular phase combination task were conducted using MATLAB 2017b (The Mathworks, Inc., Natick, MA, United States) with PsychToolBox 3.0.14 on a gamma-corrected polarized 3D monitor (27-in; D2757PH, AOC, Inc., 1,920′ × 1,080′) in a dark room. The refresh rate was 60 Hz. Polarized glasses were used during the test. The maximum luminance was set to 96.4 cd/m^2^ and was reduced to 44.8 cd/m^2^ using polarized glasses.

### Experimental design

We performed two experiments in our study. In Experiment 1, we tested the visual acuity of each eye in dichoptic and monocular conditions using the dichoptic letter chart to measure their changes in visual acuity (CVA). Then, we examined the relationship between visual acuity changes in the depth of suppression by using a binocular phase combination task, which measures the relative contribution of each eye in binocular vision. However, while the tested eye viewed the optotypes in the dichoptic-optotypes condition of this study, the corresponding region of the untested eye was presented with mean luminance. The impact of presenting mean luminance to the fellow eye on the visibility of the amblyopic eye has been under dispute (Huang et al., 2012; Zhou et al., 2014; Jia et al., 2015). Thus, in Experiment 2, we examined the effect of mean luminance on the visual acuity change by testing the visual acuity of each eye in dichoptic-optotypes, dichoptic-luminance, and monocular conditions. To achieve a better understanding of the relationship between suppression and CVA, we measured suppression using a Worth 4-dot test. Finally, we evaluated the test-retest reliability of the dichoptic letter chart.

### Dichoptic visual acuity test

**FIGURE 1:**
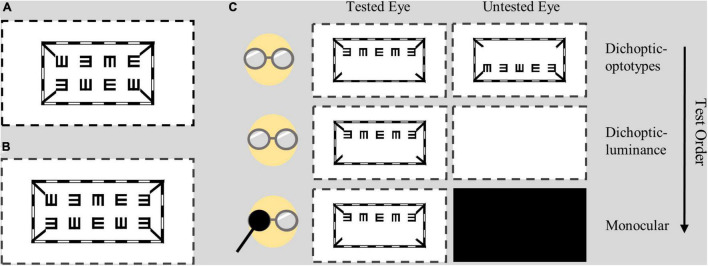
Design of the dichoptic visual acuity chart. Two lines of E letters were presented to different eyes, while the envelope and four short lines were presented to both eyes. **(A)** In Experiment 1, each line had four letters with a letter representing 0.025 logMAR. **(B)** In Experiment 2, each line had five letters with a letter representing 0.02 logMAR. **(C)** Test conditions and test order. Subjects wore polarized glasses during all test conditions. The dotted lines indicate the margin of the screen.

As shown in Figure 1, the dichoptic letter chart was comprised of a striped envelope, four short lines and a line of E letters; these were presented to the tested eye on a background of 96.4 cd/m^2^. The contrast was fixed at 100% throughout the test. The size of the letter E was drawn in logMAR form and was adjusted in a step of 0.1 logMAR through a keyboard. The test range was 1.0 logMAR to –0.1 logMAR. In Experiment 1, each row had four letters with one letter referring to 0.025 logMAR (see Figure 1A). In Experiment 2, as we wanted to better detect the difference in the three test conditions, we added five letters rather than four, each of which represented 0.02 logMAR (see Figure 1B). The directions of E were generated randomly each time. The spaces between optotypes were fixed at one E size of that line.

Subjects wore polarized glasses coupled with their own best optical correction in all testing conditions when they viewed the dichoptic visual acuity chart. The viewing distance was set to 4 m. Detailed test conditions are shown in Figure 1C. In the dichoptic-optotypes condition, the left eye was shown with the top line of Es in one plane, and the right eye was shown with the bottom line of Es in another plane; these configurations allowed the stimuli to be fused between the eyes. In the monocular condition, the visual acuity of each eye was tested by presenting one line of Es, while the untested eye was occluded with a dark opaque patch. In the dichoptic-luminance condition, the shown visual stimuli were the same as those of the monocular condition for the tested eye; however, the untested eye was presented with a blank screen that had a mean luminance of 96.4 cd/m^2^. Visual acuity was tested by presenting one or two lines of Es in monocular condition; it had no significant difference (see Supplementary Material 2).

### Suppression measurement

#### Binocular phase combination task

We used a binocular phase combination task (Ding and Sperling, 2006; Huang et al., 2009) to quantitively measure the depth of interocular suppression (Garcia-Perez and Peli, 2019; Min et al., 2021). There were two phases for every trial in this task. First, there was an alignment phase during which dichoptic crosses were shown on the screen. The subjects were asked to align the dichoptic cross into an intact fused cross to ensure that there was a proper fusion between the eyes throughout the experiment. Subsequently, a test phase followed during which two horizontal sign-wave gratings of 1 cycle/degree with a + 22.5° or –22.5° phase shift (two configurations) from the center were dichoptically presented to observers through polarized glasses at a distance of 156 cm. Before measuring suppression, we tested for sensory dominance of the normal individuals by fixing the interocular contrast at 1. While measuring suppression, we fixed the contrast of the grating presented to the non-sensory dominant eye at 100% and varied the contrast of the sensory dominant eye at different contrast ratios (i.e., 0, 0.1, 0.2, 0.4, 0.8, and 1). Observers were asked to place the flanking black reference line to the darkest position of the fused sinusoidal grating during each trial. By doing so, we were able to quantitively measure which eye was more dominant at each trial. Each trial was repeated 8 times. In total, 98 trials [2 (configurations) × 6 (contrast ratios) × 8 (repeat)] were conducted in random order. The data of perceived phases were then fitted with an attenuation gain control model (Huang et al., 2009) to obtain the interocular contrast ratio, which is where both eyes contribute equally to binocular vision. The smaller the ratio is, the larger the imbalance between eyes.

#### Worth 4-dot test

We used a Worth 4-dot (W4D) test to assess suppression in Experiment 2. During the test, subjects were asked to wear red/green anaglyph glasses and were instructed to report the number of dots and then the color of the physical white dot at the bottom at a viewing distance of 4 meters. A W4D score of 0 means no dominance (4 dots with the bottom dot yellow or red and green), 1 means partial suppression (4 dots with the bottom dot red or green), and 2 means strong suppression (only 2 or 3 dots were reported).

### Procedure

In Experiment 1, subjects performed the binocular phase combination task. They were able to take a break whenever they wanted to. Then, their visual acuity of each eye was tested using the dichoptic visual acuity chart. The order of the condition was: (1) dichoptic-optotypes condition and (2) monocular condition. The order was not randomized because we wanted to avoid the effect of monocular deprivation (Min et al., 2018); therefore, the dichoptic-optotypes condition was performed before monocular condition. In the dichoptic-optotypes condition, at each logMAR level, subjects were encouraged to report the directions of E from left to right and then from top to bottom (first left eye, then right eye). The test started at the 1.0 logMAR line and was reduced by 0.1 logMAR when the subjects reported the right directions of all 4 optotypes along the one line. If subjects failed to pass the line, the visual acuity of this eye was recorded as 1.0 logMAR minus the corresponding value of right optotypes numbers. Once the test of one eye ended, subjects were only asked to read the lines of the other eye in the subsequent logMAR levels. The performance of two eyes was recorded. Then, we covered a random eye of subject with a blank opaque patch to measure the monocular visual acuity. After finishing the monocular test of one eye, subjects had 5 min to rest under normal binocular vision. Then the other eye was tested in the monocular condition.

For Experiment 2, the procedure was similar to that used in Experiment 1 except that we measured suppression using a W4D test and inserted a dichoptic-luminance condition between the dichoptic-optotypes and monocular conditions. In the dichoptic-optotypes condition, subjects had to read at least 4 letters in the right directions if they wanted to proceed to the next line. After performing the dichoptic-optotypes condition, we measured one eye’s visual acuity of all subjects while their other eye was presented mean luminance. Subsequently, the visual acuity of the other eye was tested in the same viewing condition. On the same day, some subjects performed another testing session of dichoptic-optotypes and monocular conditions after taking a break under normal binocular vision for more than 10 min; this was included in our experimental design so that we could measure the test-retest reliability of the dichoptic letter chart.

### Statistical analysis

Data are presented as the mean ± SD unless otherwise indicated. Statistical analyses were performed using the SPSS 26.0 software package (SPSS Inc., Chicago, IL, United States). A Bland–Altman plot of test-retest reliability was drawn using the GraphPad Prism 8.4.2 software package (GraphPad Software Inc., San Diego, CA, United States). The normal distribution of data was assessed via the Shapiro–Wilk test, and the homogeneity of variance assumption was examined via the Levene test. The baseline comparability of ages in the three groups was compared using one-way analysis of variance (ANOVA). The visual acuity tested under dichoptic conditions and monocular conditions was compared using a paired *t*-test or a paired Wilcoxon rank sum test. The visual acuity change of NDE in the three groups was compared using one-way ANOVA and Fisher’s LSD *post hoc* test. Correlation relationships were computed using a Pearson correlation test or the Spearman correlation test, depending on whether the data of interest were normally or non-normally distributed, respectively. *P* < 0.05 was deemed as significant.

## Results

### Visual acuity in different conditions

**FIGURE 2:**
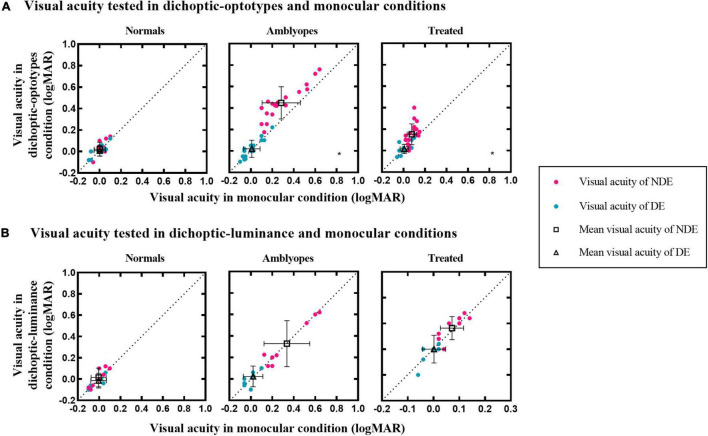
Visual acuity of the three groups in different test conditions. **(A)** Visual acuity under dichoptic-optotypes condition as a function of visual acuity under monocular condition. **(B)** Visual acuity under dichoptic-luminance condition as a function of visual acuity under monocular condition. The black dotted lines indicate the same visual acuity under two conditions. Black hollow squares and black hollow tangles represent mean visual acuity of the NDE and DE, respectively. Error bars denote the range of ± SD. A black asterisk is on the lower right of the panel which shows significant difference between two test conditions.

Figure 2 shows a plot of visual acuity for each eye in different test conditions. It shows whether there is a relationship (i.e., correlation) between visual acuity data in monocular and dichoptic conditions. To illustrate, Figure 2A shows asymmetric CVA of two eyes in treated and untreated individuals with amblyopia. The visual acuity of DE between these two conditions was not significantly different in the amblyopia (0.02 ± 0.08 vs. 0.01 ± 0.08; *t* = –1.580, *P* = 0.131) and treated groups (0.02 ± 0.04 vs. 0.01 ± 0.04; *Z* = –1.311, *P* = 0.190). However, the visual acuity of NDE in the amblyopia group was significantly reduced to 0.45 ± 0.15 logMAR in the dichoptic-optotypes condition from 0.28 ± 0.18 logMAR in the monocular condition (*t* = –9.067, *P* < 0.001). For the treated group, the visual acuity of NDE in the amblyopia group was significantly reduced to 0.15 ± 0.10 logMAR in the dichoptic-optotypes condition from 0.08 ± 0.04 logMAR in the monocular condition (*t* = –4.162, *P* = 0.001). There was no difference between these two conditions in the normal observers (NDE: *t* = –1.739, *P* = 0.104; DE: *t* = –0.653, *P* = 0.524).

To examine whether the visual acuity change in the dichoptic-optotypes condition was caused by the mean luminance of the fixing region of the untested eye, we compared the visual acuity results between the dichoptic-luminance condition and monocular condition (see Figure 2B). Visual acuity did not change in dichoptic-luminance and monocular conditions for each eye of the three groups (Normal group: *Z* = –1.289, *P* = 0.197 for NDE and *Z* = –0.816, *P* = 0.414 for DE; Amblyopia group: *t* = 0.414, *P* = 0.691 for NDE and *Z* = –0.365, *P* = 0.715 for DE; Treated group: *t* = –1.246, *P* = 0.244 for NDE and *t* = 0.287, *P* = 0.780 for DE).

### The visual acuity change of non-dominant eye

**FIGURE 3:**
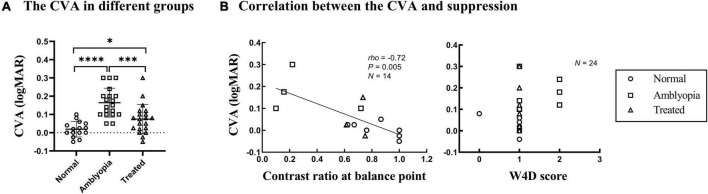
CVA in the three groups and correlation with suppression. Each dot represents the results of one patient. **(A)** The CVA in three groups. ^****^P < 0.0001; ^***^P < 0.001; *P < 0.05. **(B)** CVA as a function of the contrast ratio at the balance point tested by the binocular phase combination task in Experiment 1 and the W4D scores tested in Experiment 2. The greater the contrast ratio at the balance point is close to 1, the more balanced or less suppressed.

CVA of NDE in the dichoptic-optotypes condition and monocular condition (CVA = visual acuity of NDE in the dichoptic-optotypes condition—visual acuity of NDE in the monocular condition) were computed as an index to represent the impact of suppression on visual acuity. As shown in Figure 3A, CVA in three groups was significantly different from each other (*F* = 18.118, *P* < 0.001). The CVA of the amblyopia group was 0.16 ± 0.08 logMAR, which was more than 0.08 ± 0.08 logMAR in the treated group and 0.02 ± 0.04 logMAR in the normal group (both *P* < 0.001 in *post hoc* test). A significant difference was also found between the treated and normal groups (*P* = 0.026 in *post hoc* test). The ages of the three groups were not significantly different (*F* = 1.093, *P* = 0.343).

Figure 3B shows plots of the CVA as a function of the depth of suppression tested; the data were obtained using the binocular phase combination task and W4D test. A significant negative correlation was found between the CVA and the interocular contrast ratio at the balance point (Spearman’s *rho* = –0.72, *P* = 0.005). This result indicates that there is a positive correlation between the CVA and the depth of suppression. However, no significant relationship was found between CVA and suppression tested by the W4D test (Spearman’s *rho* = 0.380, *P* = 0.067).

### Test-retest reliability

**FIGURE 4:**
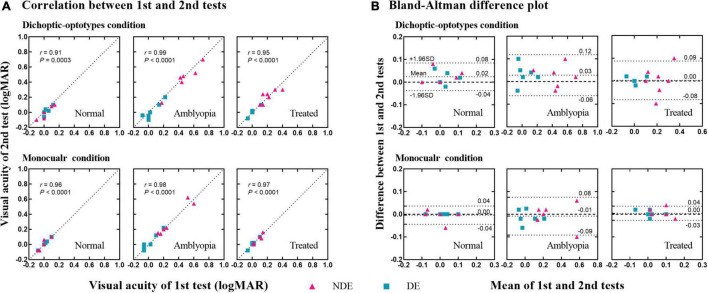
Test-retest reliability of the dichoptic visual acuity chart. **(A)** Correlation between the 1st and 2nd tests. The dotted lines indicate the line of equality (1st test = 2nd test). **(B)** Difference in visual acuity between the 1st and 2nd tests (1st test–2nd test) as a function of the mean value of the two tests [(1st test + 2nd test)/2]. Three dotted lines indicate the 95% upper limits of agreement, the bias, and the 95% lower limits of agreement, respectively.

We retested the visual acuity in dichoptic-optotypes and monocular conditions in 18 subjects (5 normal, 6 amblyopes, and 7 treated) to assess the test-retest reliability of the novel dichoptic visual acuity test in different groups. Figure 4A shows the plot of the visual acuity of each eye of the second test against the visual acuity measured from the first test. The correlation coefficients were greater than 0.90 for every group under each condition. The correlation coefficients were 0.91 (*P* = 0.0003) in dichoptic-optotypes condition and 0.96 (*P* < 0.0001) in monocular condition for normal group, 0.99 and 0.98 (both *P* < 0.0001) for amblyopia group, and 0.95 and 0.97 (both *P* < 0.0001) for treated group. Figure 4B shows a Bland–Altman difference plot of two measurements. The differences (first test–second test) are plotted against the mean values for each subject. The mean differences between the first and second tests and the 95% confidence interval (CI) limits of agreement of the normal group were 0.02 (95% CI, –0.04 to 0.08) in dichoptic-optotypes condition and 0.00 (95% CI, –0.04 to 0.04) in monocular condition. Those of the amblyopic group were 0.03 (95% CI, –0.06 to 0.12) and –0.01 (95% CI, –0.09 to 0.08). Those of the treated group were 0.00 (95% CI, –0.08 to 0.09) and 0.00 (95% CI, –0.03 to 0.04), respectively. The proportion of visual acuity difference between two measures that fell within less than 1 line (0.1 logMAR) was 93%, suggesting a robust test-retest reliability.

## Discussion

In this study, using the novel dichoptic visual acuity chart that exhibits a robust test-retest reliability, we found that the visual acuity of the amblyopic eye and the previous amblyopic eye decreased when the other eye (i.e., dominant eye) viewed optotypes but not mean luminance. Also, the magnitude of decreased visual acuity was correlated with the depth of interocular suppression.

We confirmed what has already been shown in previous studies: the visual acuity of the amblyopic eye was reduced in the dichoptic condition. The maximal extent of reduction was less than that in the study of von Noorden and Leffler (1966) in which the difference in visual acuity in the monocular condition and dichoptic condition ranged from 0 to 5 lines; however, there were 0–3 lines in this study. This may be because some of the amblyopic participants in the study of Narasimhan et al. (2012) had strabismus, which could exhibit more severe suppression than anisometropic amblyopia without strabismus.

Measurement of visual acuity of the amblyopic eye has been the standard way to diagnose and track the recovery of amblyopia in the clinic (Mintz-Hittner and Fernandez, 2000; Stewart et al., 2003). However, this approach is strictly monocular and does not reflect the binocular mechanism. Furthermore, amblyopia could be due to both monocular attenuation of the amblyopic eye and imbalanced interocular suppression—a binocular process—from the fellow eye to the amblyopic eye (Huang et al., 2011). Our results indicate that visual acuity of the amblyopic eye or the previous amblyopic eye was different depending on the visual state of the other eye. For instance, when the amblyopic eye was tested while the fellow was occluded with an opaque patch, the visual acuity was seemingly intact, thereby indicating that suppression was minimized. However, we found that the visual acuity of the amblyopic eye was worse when the fellow eye was open. The visual acuity change tested in our study reflects the binocular mechanism because our results showed that this residual suppression in treated amblyopia has functional significance in visual acuity.

Our results indicate that the dichoptic letter chart has a good test-retest reliability. Although this tool does not measure the depth of suppression directly, it can quantitively show the visual acuity change caused by suppression, which is correlated to its depth. Furthermore, the dichoptic visual acuity chart used here is intuitive and easy to complete for children. In future, we suggest that investigators consider using the change in visual acuity (as measured with the dichoptic visual acuity chart) between dichoptic-optotypes condition and monocular condition as an outcome measurement so that they could better show the treatment efficacy of amblyopia.

As the fellow eye was fixed at a region of mean luminance while the amblyopic eye was fixed at optotypes in this study, we tested whether the visual acuity change was caused by mean luminance. We have the same result as Vedamurthy et al. (2007) and Zhou et al. (2014) that mean luminance in the fellow eye does not affect the visibility of the amblyopic eye. This confirms that the reduced visual acuity observed in our study might not be caused by mean luminance. In contrast, Jia et al. (2015) found that the mean luminance stimulus of the fellow eye reduced the contrast sensitivity of the amblyopic eye. Lai et al. (2011) also found that the visual acuity and contrast sensitivity of the amblyopic eye were reduced when the fellow eye was partially patched (by presenting square mean luminance to the fellow eye). Several factors may account for these discrepancies: (1) The measuring targets: optotypes were used as measuring targets of the tested eye in our study and those of Vedamurthy et al. (2007) and Zhou et al. (2014). These optotypes had different bandwidths from sine-gratings used in the study of Jia et al. (2015); (2) interocular contrast difference: 78% contrast at the edge of square mean luminance in the fellow eye and 20% Weber contrast optotypes in the amblyopic eye were used in the study of Lai et al. (2011). Presenting optotypes to the untested eye rather than mean luminance could be more useful if one was interested in measuring the visual acuity change of the tested eye caused by suppression. This would be because two eyes would be presented with the same luminance and contrast level.

### Limitations

Our novel visual acuity chart could be limited by the possible influence of the crowding effect (Stuart and Burian, 1962; Levi, 2008). The crowding effect can be induced when the flankers and target are presented to different eyes (Flom et al., 1963). For example, the presentation of two lines of Es in dichoptic-optotypes condition and one line of Es in monocular condition could produce different crowding effects in amblyopia. This could also reduce the tested visual acuity under dichoptic-optotypes condition. If the crowding effect impacts the outcome, the visual acuity that was tested using two lines of Es should be worse than that tested using one line of Es. We conducted a control experiment (see Supplementary Material) and found no significant difference in visual acuity when it was tested using one line and two lines of Es. Thus, the crowding effect may not influence our conclusions. The crowding effect is found more in amblyopia with strabismus (Hess et al., 2001; Hariharan et al., 2005) but less in pure anisometropic amblyopia (Bonneh et al., 2004; Greenwood et al., 2012). In this study, our patients were, or used to be anisometropic amblyopia, implying that the crowding effect could be quite minimal.

If one is interested in using the dichoptic visual acuity chart to examine amblyopes with strabismus, one should account for the crowding by presenting two lines of Es to the tested eye under monocular condition.

## Conclusion

In conclusion, our study suggests that a dichoptic visual acuity chart with optotypes presented to the untested eye can be a feasible and reliable option to measure the impact of suppression on visual acuity in both the laboratory and the clinic for studying and treating amblyopia.

## Data availability statement

The raw data supporting the conclusions of this article will be made available by the authors, without undue reservation.

## Ethics statement

The studies involving human participants were reviewed and approved by the Ethics Committee on Biomedical Research of West China Hospital of Sichuan University. Written informed consent to participate in this study was provided by the participants’ legal guardian/next of kin.

## Author contributions

BZ contributed to the study design, data acquisition, analysis, interpretation, and manuscript writing and revision. ML contributed to the study design, experimental equipment, data acquisition, interpretation, and manuscript writing and revision. LL contributed to the study design, experimental equipment, data interpretation, and approval of the final version for publication. All authors contributed to the article and approved the submitted version.

## Conflict of interest

The authors declare that the research was conducted in the absence of any commercial or financial relationships that could be construed as a potential conflict of interest.

## Publisher’s note

All claims expressed in this article are solely those of the authors and do not necessarily represent those of their affiliated organizations, or those of the publisher, the editors and the reviewers. Any product that may be evaluated in this article, or claim that may be made by its manufacturer, is not guaranteed or endorsed by the publisher.
