# Supplementary material for: Measuring the impact of suppression on visual acuity in children with amblyopia using a dichoptic visual acuity chart
Source: Front Neurosci. 2022 Jul 15;16:860620. doi: 10.3389/fnins.2022.860620 (PMC9334724; doi:10.3389/fnins.2022.860620)
Supplement: Supplementary file 1 [file Table_1.DOCX]

Supplementary Material 1

**Supplementary Table 1. Clinical Details of the Participants.**

| **No.** | **Age/Sex** | **Type** | **Refraction of RE** | **Refraction of LE** | **VA**  **(RE/LE)** | **NDE** | **History** |
| --- | --- | --- | --- | --- | --- | --- | --- |
| 1 | 11/M | N | -1.75×180 | -2.00×175 | 0.00/0.02 | LE | Glasses |
| 2 | 11/M | N | PL | PL | -0.10/-0.06 | LE | NA |
| 3 | 13/F | N | +1.00/-3.50×180 | +1.25/-3.50×180 | -0.08/-0.10 | RE | Glasses |
| 4 | 10/F | N | +3.00×80 | +2.50×100 | 0.10/0.06 | RE | Glasses |
| 5 | 8/M | N | +1.50/-3.00×180 | +1.50/-2.50×180 | -0.06/0.00 | LE | Glasses |
| 6 | 9/M | N | +2.00/+1.50×90 | +1.75/+1.50×90 | 0.00/-0.04 | RE | Glasses |
| 7 | 7/F | N | +2.50/+1.25×95 | +2.00/+0.75×85 | 0.10/0.00 | RE | Glasses |
| 8 | 13/F | N | -4.25/-1.00×180 | -2.50/-1.25×180 | -0.08/-0.10 | RE | Glasses |
| 9 | 7/F | N | +0.50 | +0.50 | 0.00/0.00 | LE | NA |
| 10 | 7/F | N | +0.50/+1.25×95 | +0.25/+1.75×90 | -0.06/-0.20 | RE | Glasses |
| 11 | 7/F | N | +0.50 | +0.50/-0.25×180 | -0.06/-0.08 | RE | NA |
| 12 | 7/M | N | +2.75/-3.50×180 | +3.00/-4.00×180 | 0.06/0.04 | RE | Glasses |
| 13 | 11/M | N | -1.00/-0.50×15 | -1.25/-1.00×165 | -0.10/0.00 | LE | Glasses |
| 14 | 10/F | N | +1.25/-2.25×175 | +2.25/-3.25×180 | 0.06/-0.04 | RE | Glasses |
| 15 | 8/M | N | +1.75/+2.00×95 | +1.75/+2.50×85 | -0.10/-0.10 | RE | Glasses |
| 16 | 9/M | A | PL | +4.00/+1.25×90 | 0.00/0.20 | LE | Glasses+patching |
| 17 | 7/M | A | +2.00 | +5.00/+1.00×90 | 0.00/0.40 | LE | Glasses+patching |
| 18 | 8/F | A | +3.75 | +0.75 | 0.20/0.00 | RE | Glasses+patching |
| 19 | 9/M | A | PL | +2.50 | 0.10/0.20 | LE | Glasses+patching |
| 20 | 14/M | A | -0.75×180 | +2.50/+1.00×180 | -0.08/0.4 | LE | Glasses+patching |
| 21 | 7/M | A | +1.75/+0.50×180 | +4.25/+1.25×180 | 0.00/0.38 | LE | Glasses+patching |
| 22 | 9/M | A | PL | +0.75/-2.50×25 | 0.00/0.34 | LE | Glasses+patching |
| 23 | 10/F | A | +0.75/-2.50×25 | +0.75/-0.75×180 | 0.60/0.00 | RE | Glasses+patching |
| 24 | 7/F | A | +3.75/+1.25×120 | +5.25/+1.50×90 | 0.06/0.14 | LE | Glasses+patching |
| 25 | 5/M | A | PL | +2.00/+0.75×80 | 0.06/0.26 | LE | Glasses+patching |
| 26 | 8/F | A | +6.50/-1.50×5 | +4.75/-1.50×175 | 0.30/0.02 | RE | Glasses+patching |
| 27 | 13/M | A | -2.25/-1.00×180 | +2.50/-1.50×175 | -0.08/0.20 | LE | Glasses |
| 28 | 6/M | A | +1.25 | +4.75/-0.75×40 | -0.06/0.20 | LE | Glasses+patching+training |
| 29 | 15/F | A | +1.00/+2.75×75 | -0.50/+2.50×90 | 0.14/-0.10 | RE | Glasses+patching+training |
| 30 | 13/M | A | +4.75/+1.75×90 | +0.50/+1.00×70 | 0.56/-0.10 | RE | Glasses |
| 31 | 10/F | A | PL | +5.00/+2.75×80 | -0.08/0.80 | LE | Glasses+patching+training |
| 32 | 5/M | A | +1.25 | +5.50/+0.50×100 | 0.14/0.52 | LE | Glasses+patching |
| 33 | 6/M | A | PL | +1.75 | 0.10/0.15 | LE | Glasses+patching |
| 34 | 9/F | A | +5.00/+1.50×80 | +6.50/+1.50×100 | -0.06/0.14 | LE | Glasses+patching |
| 35 | 8/M | T | +0.75/+0.50×90 | +2.50/+0.50×90 | 0.00/0.00 | LE | Glasses+patching |
| 36 | 6/M | T | +3.00/+0.50×5 | PL | 0.10/0.00 | RE | Glasses+patching |
| 37 | 7/M | T | +0.50×90 | +1.50/+2.00×90 | -0.06/0.10 | LE | Glasses+patching |
| 38 | 7/M | T | +2.50/+1.50×75 | +1.00/+1.00×85 | -0.10/0.00 | LE | Glasses+patching |

(continued)

**Supplementary Table 1. Continued**

| **No.** | **Age/Sex** | **Type** | **Refraction of RE** | **Refraction of LE** | **VA**  **(RE/LE)** | **NDE** | **History** |
| --- | --- | --- | --- | --- | --- | --- | --- |
| 39 | 15/F | T | +1.00/+0.50×90 | +2.00/+1.50×90 | 0.04/0.10 | LE | Glasses+patching |
| 40 | 9/F | T | +5.75/+1.00×70 | +7.25/+1.25×90 | -0.10/0.02 | LE | Glasses+patching |
| 41 | 6/F | T | -3.75×180 | +1.50/-4.50×180 | 0.08/0.10 | LE | Glasses+patching |
| 42 | 11/F | T | +5.25/+1.00×180 | +3.00/+0.75×175 | 0.06/-0.08 | RE | Glasses+patching |
| 43 | 6/F | T | +2.50/+0.50×90 | +4.75/+1.25×90 | 0.00/0.04 | LE | Glasses+patching |
| 44 | 6/F | T | PL | +1.50/+0.50×165 | 0.10/0.10 | LE | Glasses+patching |
| 45 | 9/F | T | +1.50/-2.25×170 | +1.75/-3.25×180 | 0.10/0.10 | LE | Glasses+patching+training |
| 46 | 10/F | T | +1.75/+1.75×90 | +1.75×90 | 0.04/-0.04 | RE | Glasses+patching+training |
| 47 | 7/M | T | +2.50/+1.25×90 | +4.50/+1.75×90 | 0.00/0.10 | LE | Glasses+patching |
| 48 | 8/M | T | +4.25/+0.50×75 | +1.00/+0.50×90 | 0.10/0.00 | RE | Glasses+patching |
| 49 | 7/F | T | +3.25/+0.50×85 | +1.25/+0.50×90 | 0.10/0.00 | RE | Glasses+patching |
| 50 | 5/M | T | +1.75/+0.50×60 | +5.25/+1.00×120 | 0.10/0.10 | LE | Glasses+patching+training |
| 51 | 8/M | T | +3.00/-0.50×175 | +5.25/-1.25×180 | 0.00/0.10 | LE | Glasses+patching |
| 52 | 5/F | T | +2.00 | +3.50/-0.50×20 | -0.04/0.02 | LE | Glasses+patching |
| 53 | 14/F | T | +0.50/+1.75×90 | +4.00/+2.00×90 | -0.16/0.02 | LE | Glasses+patching |
| 54 | 6/M | T | +1.50×95 | +3.50×85 | 0.00/0.10 | LE | Glasses+patching+training |

*F, female; M, male; RE, right eye; LE, left eye; VA, visual acuity (logMAR); NDE, non-dominant eye; PL, plano; N, normal; A, amblyopia; T, treated amblyopia.*
