# Supplementary material for: Measuring the impact of suppression on visual acuity in children with amblyopia using a dichoptic visual acuity chart
Source: Front Neurosci. 2022 Jul 15;16:860620. doi: 10.3389/fnins.2022.860620 (PMC9334724; doi:10.3389/fnins.2022.860620)
Supplement: Supplementary file 2 [file Data_Sheet_1.docx]

Supplementary Material 2

# To examine if presenting one or two lines of Es could differentially influence visual acuity, we compared the VA results tested in monocular condition with one or two lines of Es.

# 16 unilateral amblyopia (7.31 ± 0.60 years old; 11 pure anisometropic amblyopia, 5 strabismic amblyopia with or without anisometropia) and 11 treated anisometropic amblyopia (7.18 ± 0.42 years old) were tested. Written informed consent was obtained from the patients’ guardians or parents. They were presented with one line and then two lines of Es to a random eye, with the other eye occluded (see Supplementary Figure 1). Stimuli were presented through polarized glasses. Subjects were told to report only one line (refer to the position of their visible line in dichoptic-optotypes condition) in the 2-lines condition. After a rest for 5 minutes, the other eye was tested in the same way. Visual acuity tested in these two conditions was compared using a paired t-test.

# Results of different eyes were shown in Supplementary Figure 2. For the amblyopia subjects, visual acuity tested by one or two lines of Es had no significant difference in the dominant eye (*t* = 1.100, *P* = 0.289) or non-dominant eye (*t* = 0.522, *P* = 0.609). Also, no significant difference was found in the dominant eye (*t* = 0.199, *P* = 0.846) and non-dominant eye (*t* = -0.671, *P* = 0.518) of the treated unilateral amblyopia. This result shows that presenting one or two lines of Es to the same eye would not differentially influence the visual acuity.


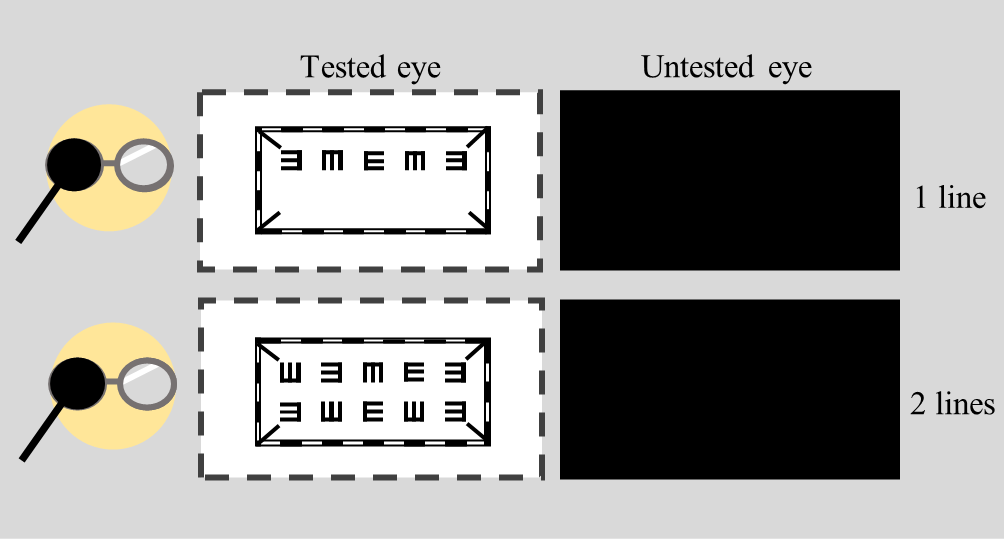


**Supplementary Figure 1.** Visual acuity test using one or two lines of Es. Subjects were told to report only one line (refer to the position of their visible line in dichoptic-optotypes condition) in the 2-lines condition.


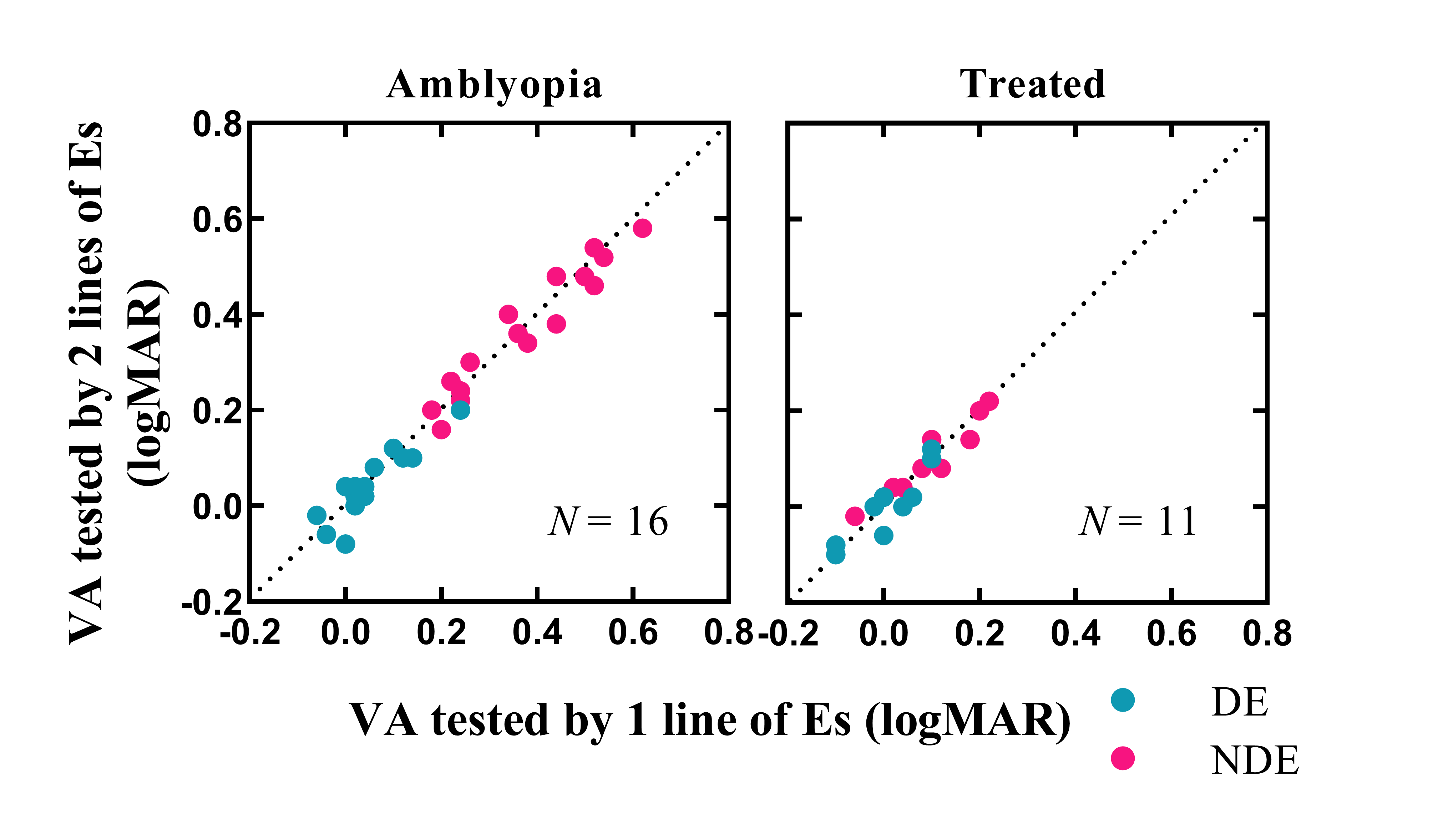


**Supplementary Figure 2.** Visual acuity tested using one line and two lines of Es. The black dotted lines indicate the same visual acuity tested by two ways. DE, the dominant eye; NDE, the non-dominant eye.
